# Vitamin D status of older adults of diverse ancestry living in the greater Toronto area

**DOI:** 10.1186/1471-2318-13-66

**Published:** 2013-07-01

**Authors:** Jaime K Ginter, S Krithika, Agnes Gozdzik, Heather Hanwell, Susan Whiting, Esteban J Parra

**Affiliations:** 1 Faculty of Humanities and Social Sciences, Sheridan College, 1430 Trafalgar Rd, Oakville, ON L6H 2L1, Canada; 2 Trent University Archaeological Research Centre (TUARC), Trent University Peterborough, Peterborough, ON K9J 7B8, Canada; 3 Department of Anthropology, University of Toronto at Mississauga, 3359 Mississauga Road North, Mississauga, ON L5L 1C6, Canada; 4 Centre for Research on Inner City Health, The Keenan Research Centre in the Li Ka Shing Knowledge Institute of St. Michael’s Hospital, Toronto, ON M5B 1W8, Canada; 5 Department of Neurosciences and Mental Health, Hospital for Sick Children, University of Toronto, Toronto, ON M5G 1X8, Canada; 6 College of Pharmacy and Nutrition, University of Saskatchewan, 110 Science Place, Saskatoon, SK S7N 5C9, Canada

**Keywords:** Vitamin D, Serum 25(OH)D concentration, Older adults, Diverse ancestry, Canada, Community-dwelling

## Abstract

**Background:**

Physiological and lifestyle factors put older adults at an increased risk of vitamin D insufficiency and resulting negative health outcomes. Here we explore the vitamin D status in a sample of community dwelling older adults of diverse ancestry living in the Greater Toronto area (GTA).

**Methods:**

Two hundred and twenty-four (224) adults over 60 years of age were recruited from the Square One Older Adult Centre, in Mississauga, Ontario. Circulating 25-hydroxyvitamin D (25(OH)D) concentrations were measured from dried blood spot cards. Dietary and supplemental intakes of vitamin D were assessed via questionnaires. Skin pigmentation was assessed quantitatively by measuring melanin levels using a reflectometer.

**Results:**

The mean 25(OH)D concentration in the total sample was 82.4 nmol/L. There were no statistically significant differences in serum 25(OH)D concentrations, supplemental or dietary vitamin D intakes between the three major ancestral groups (East Asians, Europeans and South Asians). Females had significantly higher 25(OH)D concentrations than males (84.5 nmol/L vs. 72.2 nmol/L, p = 0.012). The proportion of participants with 25(OH)D concentrations below 50 nmol/L and 75 nmol/L were 12.1%, and 38.8%, respectively. The mean daily supplemental intake of vitamin D was 917 IU/day. Vitamin D intake from supplements was the major factor determining 25(OH)D concentrations (p < 0.001).

**Conclusions:**

Mean concentration of 25(OH)D in a sample of older adults of diverse ancestry living in the GTA exceeded 80 nmol/L, and there were no significant differences in 25(OH)D levels between ancestral groups. These results sharply contrast with our recent study focused on young adults of diverse ancestry living in the same geographic area, in which we found substantially lower 25(OH)D concentrations (mean 39.5 nmol/L), low supplemental vitamin D intake (114 IU/day), and significant differences in 25(OH)D levels between ancestral groups. High daily intake of supplemental vitamin D in this sample of older adults likely accounts for such disparate findings with respect to the young adult sample.

## Background

Vitamin D plays a key role in bone development and mineralization, and it is also involved in the regulation of cell growth and immune function [1-4]. Physiological and lifestyle factors put older adults at an increased risk of vitamin D insufficiency and the resulting negative health outcomes. Cutaneous vitamin D synthesis is less efficient in older individuals due to the age-associated decline of concentrations of 7-dehydrocholesterol, which is the key precursor for the synthesis of vitamin D through the action of ultraviolet B (UVB) light in the skin [5-7]. Additionally, factors such as reduced mobility, and limited outdoor exposure, may also negatively influence vitamin D status – as measured by 25-hydroxyvitamin D (25(OH)D) levels – in some older individuals [8-13].

Numerous studies have reported that low 25(OH)D levels are associated with osteoporosis and fractures in the elderly [14,15], and these are some of the most important health concerns for this age group: osteoporosis is the suspected cause of over 300,000 hip fractures in the United States, and 24,000 hip fractures in Canada every year [16]. Long term care and health costs associated with osteoporosis-related fractures have been estimated at $13.8 billion in the United States in 1994 and $1.3 billion in Canada in 1993 [16]. Additionally, there is evidence that vitamin D supplementation may provide some benefit for physical performance in older persons [15].

Information about the vitamin D status of older adult Canadians of diverse ancestry is limited. Available studies have focused on European populations [13,17,18] or older adults of European ancestry [19,20], and to lesser extent African ancestry [21] residing in North America. Detailed descriptions of ancestry are often not reported in vitamin D studies, including national surveys in Canada [22] and older persons of diverse ethnicities have been found not to be included in meaningful numbers in studies exploring vitamin D status [15]. In particular, it is critical to obtain reliable information for darker-skinned individuals, who are at higher risk of vitamin D insufficiency because melanin (the main skin pigment) interferes with cutaneous production of vitamin D [22,23]. The aim of this study was to explore the wintertime vitamin D status of an understudied segment of the Canadian population: older adults of diverse ancestry. Secondly, we wanted to contrast our findings with previous studies of 25(OH)D levels in older adults [22,24-26], and a previous study of young adults of diverse ancestry living in the Greater Toronto Area (GTA) [23].

## Methods

### Participant characteristics and recruitment

Recruitment of participants for this study took place in February and early March of 2012 at the Square One Older Adult Centre (SOOAC), located in Mississauga, Ontario. The SOOAC was chosen because of its large, ethnically diverse membership (over 1000 members representing a range of ethnicities including, Afro-Caribbean, East Asian, European, and South Asian). Information about the study was disseminated to potential participants by way of information sheets posted in the centre, via the centre’s newsletter, word of mouth and at a health and wellness fair that was held at the SOOAC. Additional recruitment efforts were conducted at the SOOAC during their normal activities in the weeks leading up to the start of the study. The majority of participants were members of the SOOAC. A small number were friends or family of members. Age was the primary exclusion criteria and only individuals older than 60 years were eligible to participate in the study. Two hundred and twenty-four individuals agreed to participate. All participants were briefed on the purpose of the study and the extent of their involvement verbally and in the informed consent form. Every participant provided written consent. Approval to carry out this study was obtained from the Sheridan College Research Ethics Board. Participants were provided with the option to receive the results of their circulating 25(OH)D levels and were contacted with this information via phone or email after the study was complete.

### Data collection

Participation in the study involved one visit, which took place between April 18 and May 12, 2012. The data collection was done at the end of the winter in an effort to record the lowest (wintertime) 25(OH)D levels, as vitamin D levels are lowest in Canada during the winter months (November to March) when sufficient UVB is not available for cutaneous vitamin D synthesis. During this visit, participants were asked to complete a personal questionnaire that asked about their place of birth, languages spoken, self-reported ancestry, health status, and UVB exposure (self-reported time spent outdoors daily on average and travel to sunny destinations). Participants also filled out a food frequency questionnaire that assessed intakes of vitamin D from dietary and supplementary sources. The food frequency questionnaire had been validated in a previous study [27]. Participants also provided a few drops of blood on blood spot test sheets from which their 25(OH)D concentrations were measured (for more details, see below). The blood sample was collected by pricking the participant’s fingers with a single use spring action lancet and gently expressing a few drops of blood onto the blood spot test papers. Melanin content of the skin was measured in the inner upper right arm using a narrow band reflectometer (DSM II ColorMeter, Cortex Technologies, Hadsund, Denmark). This instrument provides quantitative estimates of melanin levels (e.g. Melanin index). Three measurements were taken in the upper inner arm and then averaged.

### Biochemical analysis

Measurement of 25(OH)D was carried out by ZRT Laboratory (Beaverton, Oregon) following standard protocols. 6-mm disks were punched from the middle of the pre-stamped blood spot area containing the dried blood spots (Wallac MultiPuncher) and reconstituted with 600 μl of deionized water. 600 μl of methanol containing internal standard (D4-25-hydroxyvitamin D3) was then added to precipitate proteins and the samples were vortexed. 900 μl of the supernatant was extracted with C18 solid phase extraction. Extracted samples were derivatized with 200 μl of 0.1 mg/ml PTAD (4-phenyl-1,2,4-triazoline-3,5-dione) at room temperature for 10 minutes. Derivatized samples were blown to dryness with nitrogen and reconstituted with 50 ul of methanol and 20 ul injected into the LC-MS/MS system (Varian). Previous studies have shown that there is a high correlation between the 25(OH)D levels obtained for the same individuals from blood spots, serum or whole blood [28,29].

**Figure 1:**
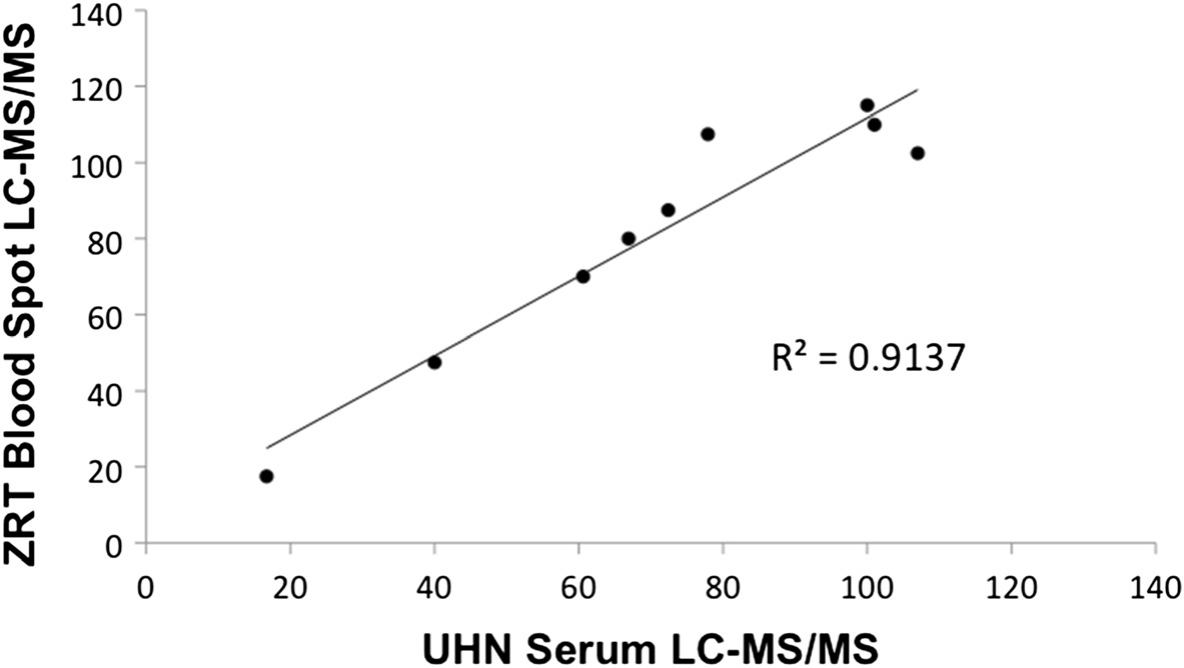
Correlation of 25(OH)D values obtained from serum and blood spots in the same samples.

**Figure 2:**
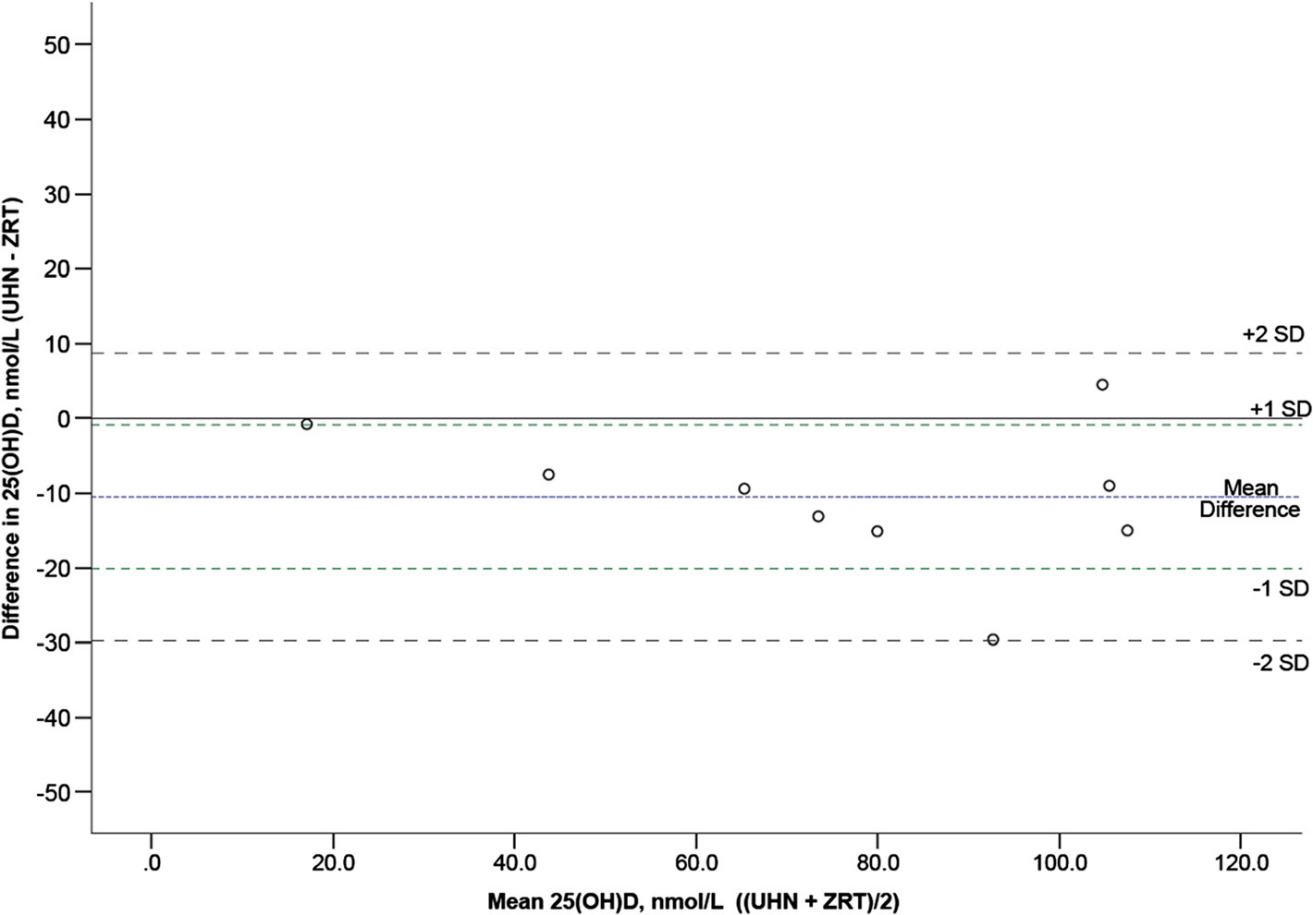
Bland-Altman plot comparing 25(OH)D estimates obtained from serum and blood spots in the same samples.

We performed an internal validation by comparing, for 10 blood spot and serum samples obtained from the same individuals, the 25(OH)D concentrations measured with the ZRT blood spot LC-MS/MS method with the values obtained using a previously described serum-based LC-MS/MS method [23]. A scatterplot showing the results of these comparisons is depicted in Figure 1. There was a very strong correlation between the 25(OH)D values obtained with both methods (r^2^ = 0.91). An analysis using a Bland-Altman plot indicated that the 25(OH)D concentrations measured in the blood spots were slightly higher than those measured in the serum samples (on average, approximately 10 nmol/L higher, Figure 2). We measured three independent blood spots for the same individual with concordant results (110, 115 and 117.5 nmol/L, respectively).

### Statistical analysis

Potential differences between groups (sex, ancestry) for 25(OH)D levels, dietary, supplemental and total vitamin D intake, and melanin index were examined using ANOVA. Linear regression was used to evaluate the major factors associated with 25(OH)D concentrations. Prior to the regression, we explored if the assumptions of the regression were met (e.g. residuals were normally distributed and no colinearity of the predictor variables was present). Potential differences in proportions (e.g. proportions of individuals classified according to commonly used 25(OH)D cutoffs used to define vitamin D status, or proportions of individuals classified according to vitamin D intake cutoffs) were explored using Fisher’s exact tests. Given the small number of participants reporting African or Other ancestry, the statistical analyses stratified by ancestry were restricted to the East Asian, European and South Asian groups. A significance level of p ≤ 0.05 was used for all tests.

## Results

### Sample characteristics

The sample consisted of 224 participants (185 females and 39 males). The average age of the participants was 72 years, with ages ranging between 60 to 90 years. Based on the information provided by the participants in the personal questionnaire, individuals were classified in five ancestral groups: African (n = 9), East Asian (n = 66), European (n = 83), South Asian (n = 64), or Other (n = 2) ancestry. Table 1 reports circulating 25(OH)D concentrations, melanin index and vitamin D intakes from food, supplements and all (total) sources for the entire sample and the main ancestral groups included in the study (East Asians, Europeans and South Asians). Table 2 presents the values of the aforementioned variables stratified by sex. The only variable that showed a significant difference between the sexes was serum 25(OH)D, with higher 25(OH)D levels recorded for females. After controlling for sex, melanin index differed significantly between the three ancestral groups. However, as described in more detail below, there were no significant differences in serum 25(OH)D concentrations, or dietary, supplemental or total vitamin D intakes between the three ancestral groups.

**Table 1:** Description of variables collected in the global sample, stratified by ancestry

	Total sample*	**African**	**East asian**	**European**	**South asian**	**Other**
N (F, M)	224 (185, 39)	9 (9, 0)	66 (51,15)	83 (77, 6)	64 (46, 18)	2 (2, 0)
Melanin Index	42.96 (34.9, 57.1)	54.8 (48.2, 61.4)	40.3 (39.1, 41.4)	39.8 (39.0, 40.7)	48.1 (46.4, 49.7)	43.2
25(OH)D (nmol/L)	82.4 (36.6, 131.3)	77.9 (64.3, 91.4)	82.1 (75.5, 88.8)	87.3 (81.3, 93.3)	77.2 (70.6, 83.8)	75
Dietary (IU/day)	168.15 (153.14, 183.15)	213.96 (133.02, 294.02)	149.25 (121.61, 176.90)	185.73 (161.51, 209.96)	157.11 (127.80, 186.41)	209.19
Supplements (IU/day)	917.41 (810.05, 1024.77)	866.67 (368.51, 1364.82)	857.58 (691.07, 1024.09)	943.37 (735.82, 1050.92	939.06 (739.45, 1138.68)	1350
Total (IU/day)	1085.57 (976.10, 1195.03)	1080.56 (620.63, 1540.48)	1006.85 (841.20, 1172.50)	1129.11 (915.70, 1342.51)	1096.19 (891.60, 1300.77)	1559

Mean values and 5th and 95th percentiles (in parentheses) are reported.

* Total sample comprises individuals of African ancestry (n = 9), East Asian ancestry (n = 66), European ancestry (n = 83), Other ancestry (n = 2), and South Asian ancestry (n = 64).

**Table 2:** Sex differences in clinical and biochemical variables

	**Female mean (n = 185)**	**Male mean (n = 39)**	**F**	**p**
25(OH)D (nmol/L)	84.5	72.2	6.432	0.012*
Melanin Index	42.9	43.2	0.082	0.775
Dietary (IU/day)	170.02	159.28	0.285	0.594
Supplements (IU/day)	958.4	723.1	2.703	0.102
Total (IU/day)	1128.41	882.36	2.845	0.093

### Vitamin D status and ancestry

The mean 25(OH)D concentration of the entire sample was 82.4 nmol/L. Circulating 25(OH)D concentrations did not significantly differ between the three ancestral groups (p = 0.081) (Table 1). Table 3 reports the proportion of individuals within the entire sample and the three ancestral groups stratified according to three commonly used 25(OH)D cutoffs: < 25 nmol/L, < 50 nmol/L, <75 nmol/L and ≥ 75 nmol/L. The majority of individuals in each ancestral group had 25(OH)D concentrations exceeding 75 nmol/L. A small proportion of participants (12.1% for the total sample and 10.6%, 10.8% and 15.6% for the East Asian, European and South Asian groups, respectively) had 25(OH)D concentrations < 50 nmol/L, which is the cutoff used by the Institute of Medicine (IOM) to define vitamin D insufficiency [15].

**Table 3:** Wintertime vitamin D status in the global sample, stratified by ancestry

	Total sample* (n = 224)	**East asian (n = 66)**	**European (n = 83)**	**South asian (n = 64)**
<25 nmol/L	3 (1.4)	2 (3.0)	0 (0.0)	1 (1.6)
<50 nmol/L	27 (12.1)	7 (10.6)	9 (10.8)	10 (15.6)
<75 nmol/L	87 (38.8)	28 (42.4)	27 (32.5)	28 (43.8)
>75 nmol/L	130 (61.0)	38 (57.6)	36 (67.5)	36 (56.3)

Absolute numbers and proportions (in parentheses) are reported.

* Total sample comprises individuals of African ancestry (n = 9), East Asian ancestry (n = 66), European ancestry (n = 83), Other ancestry (n = 2), and South Asian ancestry (n = 64).

### Vitamin D intake and ancestry

The mean daily total vitamin D intake for the entire study sample was 1086 IU/day (Table 1). The mean daily total intake was slightly higher for Europeans than for South Asians and East Asians, but the differences were not statistically significant. In the entire study sample, and also within each ancestral group, the mean daily supplemental intake of vitamin D (917 IU/day) was substantially higher than the mean daily dietary intake (168 IU/day).

Current recommendations set by the IOM [15] suggest individuals over 70 years of age should have a vitamin D intake of 800 IU/day. We divided the total sample in two groups: 1) individuals with daily vitamin D intakes < 800 IU (34.7%) and 2) individuals with daily vitamin D intakes ≥ 800 IU (65.2%). Both groups were stratified by ancestry and the aforementioned 25(OH)D cutoffs (Table 4). Almost three-quarters of individuals with daily intakes exceeding the IOM recommendations had 25(OH)D concentrations in the optimal range (> 75 nmol/L) compared to just over one third of participants who did not meet the IOM daily vitamin D recommendation. A contingency table analysis comparing the < 800 IU and ≥ 800 IU groups was statistically significant (p < 0.001).

**Table 4:** Vitamin D status of individuals with vitamin D intakes higher than 800 IU/day, stratified by ancestry

	Total sample* (n = 224)	**East asian (n = 66)**	**European (n = 83)**	**South asian (n = 64)**
**<800 IU/day**	**77**	**23**	**28**	**23**
<25 nmol/L	3 (4.0)	2 (8.7)	0 (0.0)	1 (4.3)
<50 nmol/L	24 (31.2)	6 (26.0)	8 (28.6)	9 (39.1)
<75 nmol/L	48 (62.3)	16 (69.6)	15 (53.6)	15 (65.2)
>75 nmol/L	29 (37.7)	7 (30.4)	13 (46.4)	8 (34.8)
**>800 IU/day**	**147**	**43**	**55**	**41**
<25 nmol/L	0 (0.0)	0 (0.0)	0	0 (0.0)
<50 nmol/L	3 (2.0)	1 (2.3)	1 (1.8)	1 (2.4)
<75 nmol/L	39 (26.5)	12 (27.9)	12 (21.8)	13 (31.7)
>75 nmol/L	108 (73.5)	31 (72.1)	43 (78.2)	28 (68.3)

Absolute numbers and proportions (in parentheses) are reported.

* Total sample comprises individuals of African ancestry (n = 9), East Asian ancestry (n = 66), European ancestry (n = 83), Other ancestry (n = 2), and South Asian ancestry (n = 64).

We also explored the effect of vitamin D supplements on vitamin D concentrations, by dividing the total sample into individuals who did not take supplements (23.1%) and those who did take supplements (76.9%). We stratified both groups by ancestry and the aforementioned 25(OH)D cutoffs. These values are reported in Table 5. Supplement use showed a strong relationship to vitamin D status as only one-third of participants who did not take supplements had 25(OH)D concentrations in the optimal range compared to almost three-quarters of participants who took supplements. These differences are highly significant (Fisher’s exact test p-value <0.001). See Table 5 for detailed information about the three major ancestral groups included in the analysis.

**Table 5:** Vitamin D status of individuals taking and not taking vitamin D supplements, stratified by ancestry

	Total sample* (n = 224)	**East asian (n = 66)**	**European (n = 83)**	**South asian (n = 64)**
**No Supplements**	**52**	**17**	**22**	**11**
<25 nmol/L	3 (5.8)	2 (11.8)	0 (0.0)	1 (9.1)
<50 nmol/L	19 (19.2)	5 (29.4)	8 (36.4)	6 (54.5)
<75 nmol/L	35 (67.3)	12 (70.6)	15 (68.2)	7 (63.6)
>75 nmol/L	17 (32.7)	5 (29.4)	7 (31.8)	4 (36.4)
**Supplements**	**172**	**49**	**61**	**53**
<25 nmol/L	0 (0.0)	0 (0.0)	0 (0.0)	0 (0.0)
<50 nmol/L	8 (4.7)	2 (4.1)	1 (1.6)	4 (7.5)
<75 nmol/L	52 (30.2)	16 (32.7)	12 (19.7)	21 (39.6)
>75 nmol/L	120 (69.8)	33 (67.3)	49 (80.3)	32 (60.4)

Absolute numbers and proportions (in parentheses) are reported.

* Total sample comprises individuals of African ancestry (n = 9), East Asian ancestry (n = 66), European ancestry (n = 83), Other ancestry (n = 2), and South Asian ancestry (n = 64).

### Factors affecting vitamin D status

In order to evaluate the main factors associated with 25(OH)D concentrations, a multiple linear regression was performed with 25(OH)D concentration as the dependent variable and sex, supplemental intake, dietary intake and melanin index as the independent variables. The regression analysis indicated that sex had a marginal effect on 25(OH)D levels (p = 0.052) and supplemental vitamin D intake had a strong relationship with 25(OH)D concentrations (p-value < 0.001). Dietary intake and melanin levels did not have any significant effects on 25(OH)D. The regression model revealed that just over 20% of the variation in 25(OH)D concentrations was explained by the linear combination of the variables tested. A more detailed analysis using a multi-step regression strategy indicated that intake of vitamin D supplements was responsible for about 19% of the variation in serum 25(OH)D concentrations, and a partial correlation analysis provided similar results (R^2^ = 0.194).

## Discussion

Here, we report the vitamin D status in a sample of community dwelling, active older adults (age > 60 years) of diverse ancestry living in the Greater Toronto Area (GTA). The mean 25(OH)D concentration for the entire sample was slightly higher than 80 nmol/L and we did not observe significant differences in 25(OH)D levels between the main ancestral groups analyzed in this study: Europeans, East Asians and South Asians. Perhaps more relevant than the average 25(OH)D concentrations is the proportion of participants with insufficient 25(OH)D levels. Unfortunately, there is no universal agreement with respect to which 25(OH)D cutoffs should be used to define vitamin D insufficiency, and while the IOM [15] has set this value at 50 nmol/L, many vitamin D experts and also specialty societies (e.g. Endocrine Society, the National Osteoporosis Foundation, and Osteoporosis Canada) support a cutoff of 75 nmol/L [30-32]. Using the IOM criteria, only a small number (12.1%) of our participants would be considered to have insufficient 25(OH)D levels, with little variation among the main ancestral groups (10.6-15.6%). However, using the higher cut-point of 75 nmol/L, more than a third of our total sample, and nearly half of those of South Asian and East Asian ancestry would be considered vitamin D insufficient. More than three-quarters of the individuals in the sample took vitamin D supplements, and vitamin D intake from supplements was the major factor explaining the variation in 25(OH)D concentrations in this sample. Overall, the mean supplemental intakes of vitamin D were substantially higher than the mean dietary intakes in the total sample and each of the ancestral groups.

### Comparison with previous Canadian studies

#### Young adults of diverse ancestry

It is very instructive to compare the main findings of this study with previous data available for Canada. In 2010, we published a paper describing the vitamin D status of an ancestrally diverse sample of young adults recruited in the same geographic region [23]. Table 6 reports 25(OH)D concentrations, daily vitamin D intake (total, dietary and supplementary), and percentage of participants taking vitamin D supplements, in the total samples of older and young adults, and also the three major ancestral groups (East Asia, Europe and South Asia). The mean 25(OH)D concentration in the older adult sample (82.4 nmol/L) was substantially higher than in the young adult sample (39.5 nmol/L), and this was also observed for the three ancestral groups. These differences were highly significant in all cases (p-values < 0.001 for the total sample and each ancestral group). The primary factor driving these differences in 25(OH)D concentrations was the higher vitamin D intake from supplements in the older adult sample, with respect to the young adults - 917 IU/day on average, compared to only 114 IU/day in young adults. In contrast with the sample of older adults, in which 76.8% took supplements, only 24.0% of the young adults took supplements.

**Table 6:** Comparison of vitamin D status between older adults and young adults*, stratified by ancestry

		**Total sample**	**East asian**	**European**	**South asian**
Serum 25(OH)D	Older adult	82.4 (N = 224)	82.1 (n = 66)	87.3 (n = 83)	77.2 (n = 64)
	Young Adult*	39.5 (N = 342)	33.4 (n = 99)	53.9 (n = 108)	29.2 (n = 94)
Dietary intake	Older adult	168.15	149.25	185.73	157.11
	Young Adult*	175.85	172.59	170.37	183.21
Supplemental intake	Older adult	917.14	857.58	943.37	939.06
	Young Adult*	114.88	67.68	127.39	118.94
Total intake	Older adult	1085.57	1006.85	1129.11	1096.19
	Young Adult*	290.72	240.27	297.76	302.15
Taking Supplements ^	Older adult	172 (76.8)	49 (74.2)	61 (73.5)	53 (82.8)
	Young Adult*	82 (24.0)	16 (16.2)	33 (30.6)	24 (25.5)
Not Taking Supplements ^	Older adult	52 (23.2)	17 (25.8)	22 (26.5)	11 (17.2)
	Young Adult*	260 (76.0)	83 (83.8)	75 (69.4)	70 (74.5)

* Data from Gozdzik et al. [23].

^ Absolute numbers and proportions (in parentheses) are reported.

It is possible that sample and methodological differences between the two studies may partly account for the difference in vitamin D status between the young and old adult samples. The 25(OH)D concentrations in the older adult sample were measured using a blood spot LC-MS/MS method, whereas in the young adult sample a serum-based LC-MS/MS method was used. We compared the performance of both methods in the same samples (see Methods section) and found that the blood spot method provided values that were approximately 10 nmol/L higher on average, which is much smaller than the mean differences observed in the older adult and young adult samples. Another caveat to inter-study comparisons is that the data collection in the older adult study took place in April/May, a period in which endogenous vitamin D synthesis is possible in Southern Ontario. The data collection in the young adult study took place in January/February when no synthesis was possible; these seasonal disparities between studies may also contribute to the higher 25(OH)D levels observed in the present study of older adults. Another difference between the two studies was that in the young adult study we excluded individuals who travelled to sunny locations prior to participating in the study, and this was not a criterion of exclusion in the older adult study. Mitigating concerns about this difference is the observation that, in the present older adult study, 25(OH)D concentrations did not significantly differ between participants who travelled to sunny destination during the three months prior to their involvement in the study and those who did not travel. In fact, individuals who did not travel (n = 156) reported slightly higher mean 25(OH)D levels (mean = 83.6 nmol/L) than those who did travel (n = 68, mean = 79.6 nmol/L). In summary, we conclude that the observed differences in vitamin D status between the older and young adults are largely driven by the substantially higher daily supplementary vitamin D intakes in the older adults – 917 IU/day on average, compared to only 114 IU/day in young adults.

#### Older adults

We also compared our findings with those of three Canadian studies that surveyed older adults, the Canadian Health Measures Survey (CHMS) [22,24], the Canadian Community Health Survey (CCHS) [25], and a recent report focusing on 25(OH)D levels in older individuals residing in long term care in the same geographic area where our study was conducted, Mississauga, Ontario [26].

The CHMS reported the vitamin D status in a sample of older Canadians aged 60–79 years (in addition to other age groups). This survey reflected the Canadian population having > 80% of European ancestry, but the number of participants of other ancestry groups was too low to carry out meaningful comparisons. The proportion of older adults in the CHMS study with 25(OH)D serum concentrations < 50 nmol/L (measured with the LIASON 25-Hydroxyvitamin D Total assay) was similar to the proportion observed in our study (14.4% vs. 12.1%, respectively). The mean 25(OH)D concentrations in older adults (males and females) taking supplements vs. those not taking supplements were similar in both studies: Females taking supplements had mean 25(OH)D concentrations of 89.2 nmol/L and 84.4 nmol/L in our study and the CHMS study, whereas the concentrations for females not taking supplements were 66.2 nmol/L and 61.9 nmol/L, respectively. For males taking supplements, 25(OH)D concentrations were 78.9 nmol in our study vs. 83.1 in the CHMS study, whereas males not taking supplements had lower mean concentrations of 61.5 nmol/L and 63.1 nmol/L, respectively.

The Canadian Community Health Survey [25] collected information on dietary vitamin D intake across the country. This survey did not explore specific ancestral differences in vitamin D intakes, so the mean dietary intake for our total older adult sample was used in the comparative analysis. We restricted our comparisons to the information available for two groups of older adult Ontarians (51–70 yrs, and >70 yrs). The dietary intakes ranged between 6.5 ug/day (260 IU/day) in the CCHS sample of male adults aged 51–70 yrs and 4.7 ug/day (188 IU/day) in our sample of older adult males aged 60–70 yrs. There were no significant differences in vitamin D dietary intakes estimated in our sample and the CCHS.

Finally, a recent paper by Ioannidis et al. [26] described 25(OH)D concentrations and vitamin D supplement use in approximately 100 older residents living in four long term care facilities in Mississauga. Only 49.0% (48/98) of the residents were taking vitamin D supplements. This contrasts with the proportion of older adults in our study taking vitamin D supplements, 76.8% (172/224) (p < 0.001). Ioannidis also reported that mean serum 25(OH)D concentrations in individuals not taking supplementation was 62.6 nmol/L, and the mean levels were 72.9 nmol/L, 98.9 nmol/L and 96.0 nmol/L for individuals taking vitamin D supplementation of 0–400 IU/day, 401–800 IU/day, and > 800 IU/day, respectively. Using the same cutoffs as Ioannidis, we found very similar concentrations in our sample. Participants taking no supplementation, 0–400 IU/day, 401–800 IU/day and > 800 IU/day had mean 25(OH)D concentrations of 65.0 nmol/L, 72.7 nmol/L, 83.6 nmol/L and 90.9 nmol/L, respectively. Therefore, we observe that in both studies the mean 25(OH)D concentrations increase with the amount of vitamin D supplementation, and the mean concentrations in each supplementation group are quite similar. However, the proportion of older adults in our community dwelling sample taking vitamin D supplements was substantially higher than in the older residents living in long term care facilities (76.8% vs. 49.0%, respectively). Another recent Canadian study that analyzed vitamin and mineral supplements in a long-term care residence has indicated that only 35.4% of all the residents in the facility took vitamin D supplements [33].

In general, our results show good agreement with the results of other Canadian older adult studies in terms of (i) the proportion of individuals with 25(OH)D concentrations < 50 nmol/L (our study vs. CHMS study), (ii) the 25(OH)D concentrations amongst individuals taking vitamin D supplements and those not taking supplements (our study vs. CHMS study vs. Ioannidis study), and (iii) the mean daily vitamin D dietary intakes (our study vs. CCHS study). However, the comparison of our study, in which we sampled community dwelling older adults, and two studies which sampled older adults in long term care residences [26,33], seems to indicate that the percentage of older adults living in long term care residences taking vitamin D supplements is considerably lower. This emphasizes the need to carry out studies that are more representative of the broader Canadian older adult population to explore in more detail to which extent there are differences in vitamin D supplementation between community dwelling older adults and long term care residents, and how this potential difference influence vitamin D status.

This study is not without limitations. The study participants were community dwelling, active older adults, and thus are not representative of the entire older adult population living in the GTA. Members of the SOOAC are probably more mobile and healthier than the general older adult population in this region, on account of their membership in a community organization that promotes active, healthy aging. Furthermore, it is possible that the members of the SOOAC who volunteered to participate in our study may represent those members that are interested in healthy ageing, aware of the benefits of vitamin D and actively supplementing with vitamin D. Previous studies have reported that institutionalized and home bound [8,9,12,13] older adults are at a higher risk of vitamin D deficiency than community dwelling older adults. Indeed, as described above, the comparison of our results with those of two studies focusing on older adults living in long term care residences supports this concept in that the proportion of individuals taking vitamin D supplements is much lower amongst those in long-term care vs. those in the community. We also encountered some challenges in the application of the food frequency questionnaire, some of which are common to studies using this tool to estimate dietary intakes. Some participants experienced difficulty providing accurate estimates of their average weekly diet and some individuals did not complete serving sizes for all dietary questions. Language limitations were another issue for some of the participants. Despite the SOOAC being a highly integrated, diverse centre, there were some individuals for whom English proficiency was an issue. This was especially true for one of the cultural groups, for which many members did not speak or read English to the level required for this study. This issue was resolved by translating the questionnaires into the native language and having one of the coordinators of this group translate and assist those participants who were not proficient in English with the completion of the questionnaires. Given that the vitamin D dietary intakes estimated in this study were not significantly different to those reported in other studies [22,23], it is unlikely that the aforementioned issues had a major impact on our estimates of vitamin D intake. Finally, the only criterion for exclusion in this study was age. We did not exclude participants based on other criteria, such as recent trips to sunny destinations, use of certain medications, or medical conditions, all of which may have an effect on vitamin D metabolism. As such, we feel that we were best able to obtain a broad representation of community dwelling older adults of diverse ancestry.

## Conclusions

In conclusion, in this study we observed that the mean circulating 25(OH)D concentrations in a group of community dwelling older adults of diverse ancestry living in the GTA slightly exceeded 80 nmol/L and did not significantly between the three ancestral groups (East Asians, Europeans and South Asians). In terms of the health implications of our study, the most relevant figure is the proportion of participants with suboptimal vitamin D levels. Using the IOM cutoff of 50 nmol/L, approximately 12% of the participants would have been classified as insufficient. However, using the higher cutoff of 75 nmol/L, the proportion of participants with vitamin D insufficiency would be nearly 40%. Dietary vitamin D intakes were relatively low (<200 IU/day), and, thus, insufficient to cover the daily requirements established by the IOM (600–800 IU/day) and many other health agencies. In contrast, intake of vitamin D supplements exceeded IOM recommendations and was the main determinant of 25(OH)D levels. Moreover, the prevalence of insufficiency was substantially higher amongst individuals not taking supplements, irrespective of the cutoff used. Our results indicate that taking vitamin D supplements is important to ensure optimal vitamin D levels, particularly in the Canadian winter when there is insufficient ultraviolet B radiation to cutaneously synthesize vitamin D. This is of particular relevance for older adults, who are more vulnerable to vitamin D insufficiency and deficiency than other age groups, due to a number of physiological and lifestyle factors.

## Abbreviations

SOOAC: Square One Older Adult Centre; CHMS: Canadian Health Measures Survey; CCHS: Canadian Community Health Survey.

## Competing interests

The authors declare no conflict of interests.

## Authors’ contributions

JKG co-conceived the study, participated in its design and coordination, was involved in the recruitment of participants, performed the statistical analysis, and helped to draft the manuscript. SK helped to coordinate the study, and was involved in the recruitment of participants. AG provided comparative data from the young adult vitamin D study, and helped with the interpretation of these data. HH helped with the comparisons between the serum LC-MS method and the ZRT LC-MS method, and with the interpretation of the CHMS and CCHS data. SW provided and analyzed the food frequency questionnaire, and helped with the interpretation of dietary and supplemental vitamin D intake data. EJP co-conceived the study, participated in its design, and helped to draft the manuscript. All authors read and approved the final manuscript.

## Pre-publication history

The pre-publication history for this paper can be accessed here:

http://www.biomedcentral.com/1471-2318/13/66/prepub
